# Biofumigation on Post-Harvest Diseases of Fruits Using a New Volatile-Producing Fungus of *Ceratocystis fimbriata*

**DOI:** 10.1371/journal.pone.0132009

**Published:** 2015-07-06

**Authors:** Qian Li, Lei Wu, Jianjun Hao, Laixin Luo, Yongsong Cao, Jianqiang Li

**Affiliations:** 1 Beijing Engineering Research Center of Seed and Plant Health (BERC-SPH) / Beijing Key Laboratory of Seed Disease Testing and Control (BKL-SDTC), Beijing, P. R. China; 2 Laboratory of Fruit Quality Biology, The State Agriculture Ministry Laboratory of Horticultural Plant Growth, Development and Quality Improvement, Zhejiang University, Zijingang Campus, Hangzhou, P. R. China; 3 School of Food and Agriculture, The University of Maine, Orono, ME, United States of America; The University of Wisconsin - Madison, UNITED STATES

## Abstract

A variety of volatile organic compounds (VOCs) produced by *Ceratocystis fimbriata* have strong bioactivity against a wide range of fungi, bacteria and oomycetes. Mycelial growth, conidial production, and spore germination of fungi and oomycetes were significantly inhibited after exposure to cultures of *C*. *fimbriata*, and colony formation of bacteria was also inhibited. Two post-harvest diseases, peach brown rot caused by *Monilinia fructicola* and citrus green mold caused by *Penicillium digitatum*, were controlled during a 4-day storage by enclosing wound-inoculated fruits with 10 standard diameter Petri plate cultures of *C*. *fimbriata* in a 15 L box. The fruits were freshly inoculated at onset of storage and the cultures of *C*. *fimbriata* were 6 days old. Percentage of control was 92 and 97%, respectively. After exposure to *C*. *fimbriata* VOCs, severely misshapen hyphae and conidia of these two post-harvest pathogens were observed by scanning electron microscopy, and their pathogenicity was lost or greatly reduced.

## Introduction

The development and utilization of volatile organic compounds (VOCs) from microorganisms have been of increasing interest since they are naturally produced without chemical synthesis. VOCs are mixtures of carbon-based compounds that are either vapors or highly volatiles [1]. The identified VOCs generated by fungi and bacteria are mainly primary (from the synthesis of DNA, amino and fatty acids, etc.) and secondary metabolites (from intermediates of primary metabolism) [2]. Microbial VOCs may be signaling substances for regulating and controlling certain physiological actions of some plants. The growth of *Arabidopsis thaliana* was promoted after the exposure to the volatiles from bacteria [3], and the systemic resistance in *A*. *thaliana* against *Erwinia carotovora* was induced [4]. Root length, shoot length and fresh weight were increased by treating lettuce seedlings with VOCs produced by *Fusarium oxysporum* MSA 35 [5]. Volatile compounds from plant growth-promoting fungi (PGPF) significantly reduced disease severity in *Arabidopsis* plants [6]. VOCs can attract or deter insects and other invertebrates as “semiochemicals” [1], which are defined as chemicals involved in the interactions between organisms [7]. Some volatiles have been considered as indicators of biocontamination in the food processing industry or the basis of taxonomic research [2].

Recently, fungal VOCs have been developed as green chemicals and fuel sources called “mycodiesel” [8]. Additionally, fungal VOCs have been used as biological control agents (“biofumigant”) for plant disease and pest management and have been described as eco-friendly due to a reduction in the need for application of synthetic fungicides. *Trichoderma* species have been used as biological agents for more than 80 years [9] and produce anti-microbial compounds that kill and degrade other pathogenic fungi [10], induce systemic resistance [11], and promote the growth of plants [12]. Volatile substances from *Streptomyces philanthi* inhibited the growth of *Rhizoctonia solani* on rice leaves [13]. *Bacillus subtilis* produced antagonistic volatile compounds that caused structural deformations in six pathogenic fungi, such as *Alternaria alternata*, *Cladosporium oxysporum*, *F*. *oxysporum*, *Paecilomyces lilacinus*, *P*. *variotii*, and *Pythium afertile* [14]. Compounds from *B*. *vallismortis* showed strong growth inhibition activity *in vitro* against some plant pathogens [15]. *Serratia rubidaea* generating rhamnolipids under solid-state fermentation (SSF) was used as a biocontrol agent [16]. Another successful case is *Muscodor* species, whose volatiles inhibit or kill a wide range of plant pathogenic fungi, bacteria [17], nematodes [18], insects [19] and even human pathogens [20]. These volatiles have been developed into bio-fumigants for controlling various types of plant diseases [21–23], weeds [24], nematodes [18], insects [19] and molds developing within buildings [25].

*Ceratocystis fimbriata* Ellis & Halsted is a soilborne ascomycete fungus. In addition to being a plant pathogen, it can produce a variety of VOCs [1, 26]. These volatiles are affected by the nutrition in the culture media, incubation temperature, and water activity (aw). In our preliminary study, the produced VOCs were identified by gas chromatography-mass spectrometry (HS GC-MS). Better understanding the bioactivity of VOCs from *C*. *fimbriata* not only can provide a tool for controlling post-harvest diseases, but also further reveal the relationships among the microorganisms in natural ecological system. The aim of this study is to detect the bioactivity of *C*. *fimbriata* volatiles on 16 plant pathogens *in vitro*, investigate their effect in controlling peach brown rot and citrus green mold, and probe the possible mechanisms of inhibition.

## Materials and Methods

### Ethics statement

This study took place in Seed Health Centre of China Agricultural University (SHC-CAU), Room 126, Room 117 and Room 121 of Plant Protection Building in CAU. All of the microorganisms used in this study were isolated from diseased plants or fruits, and no endangered or protected species involved.

### Isolates of fungi, oomycetes and bacteria

Our source of *C*. *fimbriata*, currently stored at the Seed Health Centre of China Agricultural University (SUC-CAU), was originally isolated from a diseased pomegranate tree in Mengzi County of Yunnan Province in China [27]. The test plant pathogens, including fungi, oomycetes and bacteria, were all isolated from diseased plant materials that were also identified and preserved in SHC-CAU (Table 1), except *Monilinia fructicola*, which was provided by Dr. L.Y. Guo, Department of Plant Pathology, CAU. The fungi and oomycetes were stored on potato dextrose agar (PDA) and carrot agar (CA) slants respectively as 5 to 7-day old cultures that were covered with paraffin oil at 4°C, and transferred to fresh PDA or CA plate and cultured in darkness at 25°C for 5 to 7 days when needed. To make conidial suspensions of test fungi, the culture of these organisms in Petri dishes were flooded with sterile water containing 0.01% Tween 80 and scraped gently with a sterilized glass rod. The suspension was then filtered through two layers of autoclaved medical gauze. A hemacytometer was used to determine the concentration of the stock suspension of conidia and based on that count the suspensions were diluted to 10^5^ conidia/mL. Bacterial strains were recovered from suspensions stored in 15% glycerol at -80°C, transferred to fresh Luria-Bertani (LB) liquid medium and cultured in darkness on a rotating shaker (120 rpm) at 28°C for 2 days. The bacterial suspensions were diluted with sterile water to a final concentration at 10^6^ CFU/mL.

**Table 1:** Test fungi, oomycetes and bacteria.

Pathogen type	Species	Host	Disease type
Fungi	*Botrytis cinerea*	Tomato	Post-harvest
	*Monilinia fructicola*	Peach	Post-harvest
	*Fusarium verticillioides*	Cabbage	Soil-borne
	*Fusarium oxysporum*	Cabbage	Soil-borne
	*Valsa mali*	Apple	Post-harvest
	*Curvularia* sp.	Dragon fruit	Post-harvest
	*Fusarium* sp.	Dragon fruit	Post-harvest
	*Penicillium italicum*	Citrus	Post-harvest
	*Penicillium digitatum*	Citrus	Post-harvest
	*Rhizoctonia solani*	Rice	Soil-borne
	*Rhizoctonia solani*	Bent grass	Soil-borne
Oomycetes	*Phytophthora sojae*	Soybean	Soil-borne
	*Phytophthora capsici*	Pepper	Soil-borne
Bacteria	*Pectobacterium carotovorum* (Gram-negative, G^-^)	Potato	Seed-borne
	*Acidovorax citrulli* (Gram-negative, G^-^)	Watermelon	Seed-borne
	*Clavibacter michiganensis* subsp. *michiganensis* (Gram-positive, G^+^)	Tomato	Seed-borne

### Bioassay for *C*. *fimbriata* VOCs against plant pathogens

**Fig 1:**
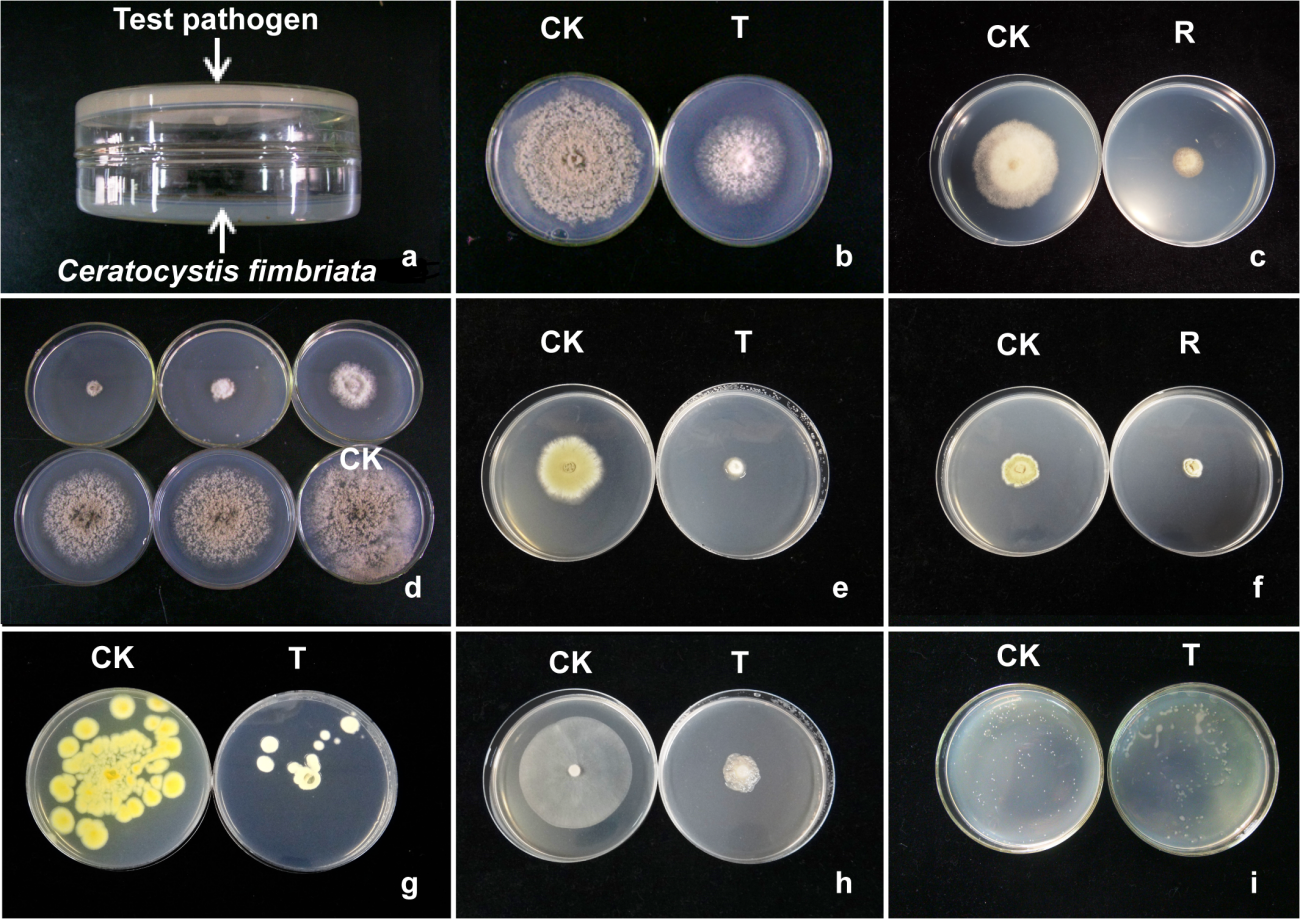
Dual-culture assay for bioactivity of volatile organic compounds produced by *Ceratocystis fimbriata*. (a) Experimental design used with the following test organisms. Plate with *C*. *fimbriata* was on the bottom and test pathogen culture was on the top plate with the culture facing down. (b) CK: *Monilinia fructicola* growing on a PDA plate for 6 days. T: *M*. *fructicola* growing on a PDA plate with 6-day exposure to the VOCs from *C*. *fimbriata*. (c) CK: *M*. *fructicola* growing on a PDA plate for 4 days. R: *M*. *fructicola* after 6-day exposure to the VOCs from *C*. *fimbriata* transferred to a fresh PDA plate and grown for another 4 days. (d) *M*. *fructicola*, exposed to 0, 1, 3, 5 and 7-day old cultures of *C*. *fimbriata* for 5 days, respectively. (e) CK: *Penicillium digitatum* growing on a PDA plate for 6 days. T: *P*. *digitatum* growing on a PDA plate with 6-day exposure to the VOCs from *C*. *fimbriata*. (f) CK: *P*. *digitatum* growing on a PDA plate for 4 days. R: *P*. *digitatum* after 6-day exposure to the VOCs from *C*. *fimbriata* transferred to a fresh PDA plate and grown for another 4 days. (g) CK: *P*. *italicum* growing on a PDA plate for 5 days. T: *P*. *italicum* growing on a PDA plate with 5-day exposure to the VOCs from *C*. *fimbriata*. (h) CK: *Phytophthora capsici* growing on a CA plate for 6 days. T: *P*. *capsici* growing on a CA plate with 6-day exposure to the VOCs from *C*. *fimbriata*. (i) CK: *Acidovorax avena* growing on a LB plate for 2 days. T: *A*. *avena* growing on a LB plate with 2-day exposure to the VOCs from *C*. *fimbriata*.

The bioactivity of *C*. *fimbriata* VOCs on test pathogens was determined by measuring the growth of test organisms with double Petri dish dual-culture (Fig 1A). The bioassay system was set up with the bottoms of two 9-cm lidless Petri dishes, which were laid in opposition and then sealed together with two layers of Parafilm (treatment, T). One plate containing PDA or CA, which was on the top of the dual-culture, was inoculated with a plug (diameter = 5 mm) of test fungi or oomycetes, or spread with 100 μL of test bacterial suspension at 10^6^ CFU/mL on LB plate. The other one, placed at the bottom, was a 6-day-old culture of *C*. *fimbriata* (diameter of colony of *C*. *fimbriata* was about 4 cm) inoculated with a plug (diameter of plug = 5 mm) that produced a certain amount of VOCs. Three replicates were conducted, and a same dual-culture system lacking *C*. *fimbriata* was used as the negative control for each pathogen. The cultures were incubated in darkness at 25°C for fungi or oomycetes, or 28°C for bacteria. The growth of the test organisms, including morphology of colony, growth of mycelia, inhibition of mycelial growth, and conidial production, was measured 5 to 7 days post inoculation (DPI) for the fungi and oomycetes as the diameter of the controls reached about 8 cm and 2 DPI for the bacteria.

$$\text{Inhibition of mycelial growth}\;(\%)=\frac{\left(\text{Colony diameter of control}(\text{cm})-0.5)-(\text{Colony diameter of treated fungi or oomycetes}(\text{cm})-0.5\right)}{\left(\text{Colony diameter of control}(\text{cm})-0.5\right)}\times 100\%$$

The effect of the treatment on conidial germination of test fungi by the volatiles produced by *C*. *fimbriata* was also observed. 100 μL of a conidial suspension of test fungi at 10^5^ conidia/mL was plated on PDA, and sealed with a 6-day-old culture of *C*. *fimbriata* as described above. An untreated by the volatiles produced by *C*. *fimbriata* was used as the negative control, with three replicates for each pathogen. The incubation conditions for these dual-cultures were the same as above, and the germination per 100 conidia was counted for each plate when most of the conidia in the control were germinated.

$$\text{Inhibited percent}\;(\%)=\frac{\text{Germination rate of control}\;(\%)-\text{Germination rate of treated fungi}\;(\%)}{\text{Germination rate of control}\;(\%)}\times 100\%$$

Next, a plug from the edge of each treated pathogen was transferred to a fresh plate (recovered treatment, R) and the growth, including colony morphology, mycelial growth, conidial production and spore germination, was compared to the untreated treatment at 5 DPI. Three replicates were carried out for each pathogen, and the incubation conditions were the same as described above. The experiment was carried out a total of twice.

In order to detect the relationship between *C*. *fimbriata* VOCs concentration and the inhibition effect, the test pathogens were inoculated with a fungus or oomycete plug (diameter = 5 mm) on PDA or CA and 100 μL of test bacteria suspension at 10^6^ CFU/mL on LB, and treated with the same bioassay system by *C*. *fimbriata* cultures of 0, 1, 3, 5 or 7 days old, which had different concentration of VOCs. The treatment with no *C*. *fimbriata* inoculated was taken as the negative control in three replicates for each concentration. These cultures were incubated in darkness at 25°C for fungi or oomycetes or 28°C for bacteria. The mycelial or colony growth was measured 5 to 7 DPI for the fungi and oomycetes as the diameter of the controls reached about 8 cm, and 2 DPI for the bacteria. The work was done twice.

### Control of peach brown rot and citrus green mold by VOCs from *C*. *fimbriata*

**Fig 2:**
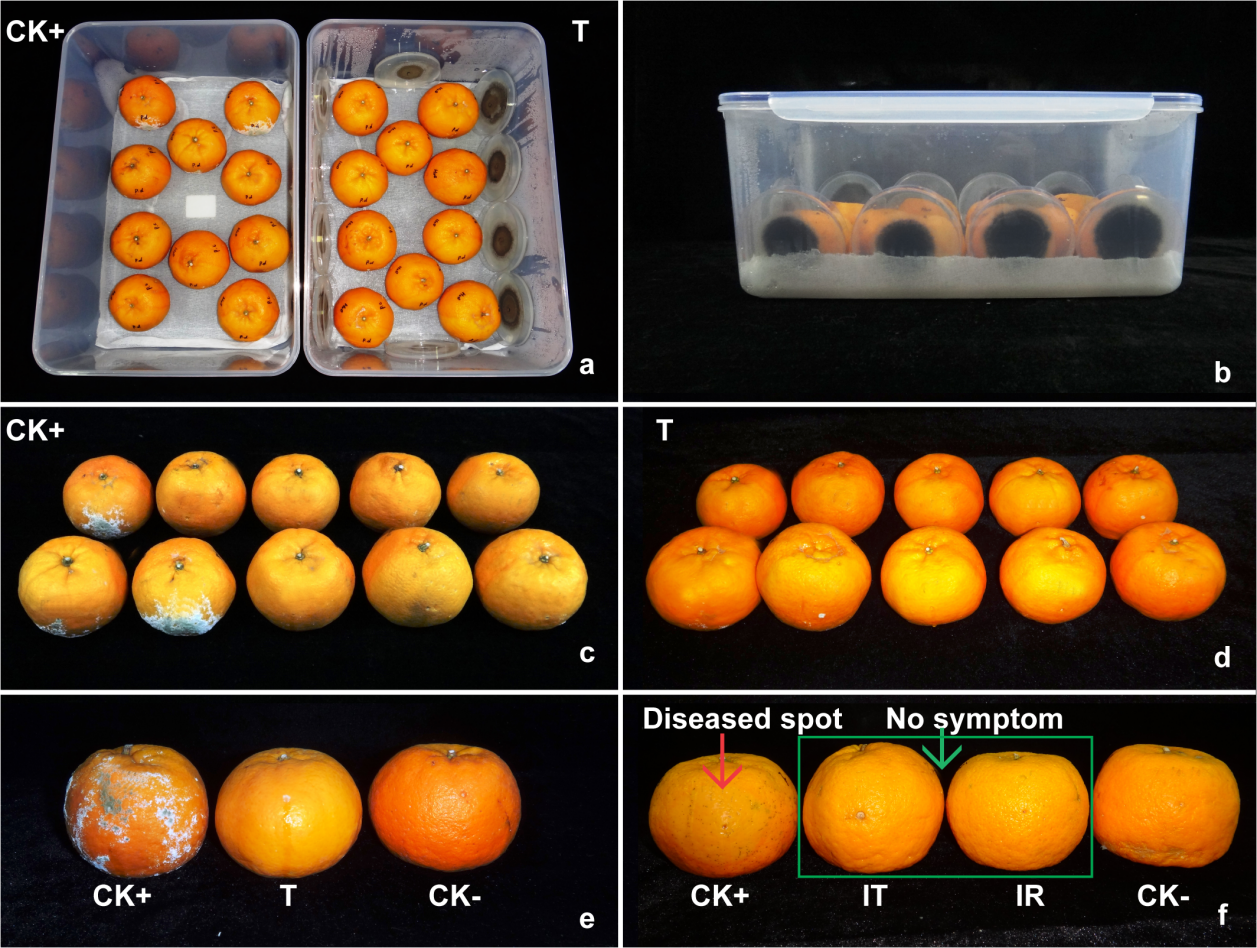
Control of citrus green mold by the VOCs from *Ceratocystis fimbriata* (4 DPI). (a-b) Experimental design for the bio-control system. (c) Citrus inoculated with 10 μL conidial suspension of *Penicillium digitatum* at 10^5^ conidia/mL without treatment. (d) Citrus inoculated with 10 μL conidial suspension of *P*. *digitatum* at 10^5^ conidia/mL and treated by the VOCs from *C*. *fimbriata*. (e) Comparison among the positive control (CK+), negative control (CK-) and treated by *C*. *fimbriata* volatiles (T). (f) Pathogenicity test of the treated and recovered *P*. *digitatum*. T (treated) = the citrus inoculated with 10 μL conidial suspension of *P*. *digitatum* at 10^5^ conidia/mL with the treatment by the VOCs form *C*. *fimbriata*. CK+ (positive control) = the citrus inoculated with 10 μL conidial suspension of *P*. *digitatum* at 10^5^ conidia/mL without treatment. CK- (negative control) = the citrus inoculated with 10 μL sterile water without treatment. IT = inoculation with treated 2 mm plug of *P*. *digitatum*. IR = inoculation with recovered 2 mm plug of *P*. *digitatum*.

Experiments were conducted with commercially grown peaches and citrus bought from Xiyuan market in Beijing. Disease- and injury- free fruits were selected and surface sanitized in 3% NaClO solution for 5 min. The fruits were rinsed with sterile water three times then placed on a clean bench for aseptic drying. The inoculation method was modified as described [21]. For each fruit, three locations were wounded at the equator of equidistance with a sterile inoculating needle. The depth of wounds was about 5 mm. Then, 10 μL of conidial suspension of *M*. *fructicola* (10^5^ conidia/mL on peach) or *P*. *digitatum* (10^5^ conidia/mL on citrus) and sterile water were pipetted into two wound locations, leaving the third wound without inoculation as a control. The inoculated fruits were placed in 15 L plastic boxes with two layers of autoclaved medical gauze, soaked with 100 mL of sterile water, placed at the bottom to moisturize and prevent rolling (Fig 2A and 2B). The effect of exposure to *C*. *fimbriata* volatiles was detected by placing a specific number of 6-day-old (inoculated with a plus, diameter = 5 mm) *C*. *fimbriata* cultures in the boxes. The boxes were placed in an illuminated incubator at 25°C after being closed with fitting plastic lids. The control consisted of inoculated fruits in boxes without inoculated *C*. *fimbriata* cultures. The effect of application time for the treatment, VOCs dose and treatment period was determined with the treatment by various amounts of inoculated *C*. *fimbriata* cultures per box or different treatment periods: immediately, 24 h, or 48 h, respectively, after inoculation on the fruits. The infected number, lesion size, and control effect were measured and calculated after 4-day treatment. Each experiment was conducted in a completely randomized design with three replicate boxes with 10 fruits in each. For lesion size measurement, 10 fruits were randomly selected from the three boxes per each treatment. $\text{Lesion size}\;({\text{mm}}^{2})=\pi \times \frac{\text{a}}{2}\times \frac{\text{b}}{2}$, a: the length of disease spot (mm), b: the width of diseases spot (mm).

$$\text{Control effect}\;(\%)=\frac{\text{Lesion size of untreated fruits}\;({\text{mm}}^{2})-\text{Lesion size of treated fruits}\;({\text{mm}}^{2})}{\text{Lesion size of control}\;({\text{mm}}^{2})}\times 100\%.$$

The experiment was repeated.

### Morphological change of the treated pathogens

The conidial and mycelial morphology of treated and recovered *M*. *fructicola* and *P*. *digitatum* was observed by a scanning electron microscope (SEM, HITACHI S-3400N) equipped with the control software HITACHI S-3400N Scanning Electron Microscope, provided by Functional Genomics Centre of platform 985, CAU, with normal cultures as controls. The PDA medium, with treated mycelia and conidia on its surface, was cut into small pieces (5 mm×5 mm) and fixed with 2.5% glutaraldehyde solution at 4°C overnight. The fixed treated mycelia and conidia with PDA medium was rinsed with phosphate buffer solution (0.1M PBS, pH 7.2) for 20 min, three times. The samples were then dehydrated in an ethanol gradient (30%, 50%, 70%, 80%, 90%, and 100% ethanol), three times for 20 min at each concentration. After the substitution treated with isoamyl acetate three times for 20 min each time, the samples were critical-point dried with CO_2_ in an HCP-2 critical-point dryer (Hitachi, Japan) for 8 hours. Then, each sample was placed onto a metal stub with double-sided carbon tape and sprayed with gold ion sputter (EIKO IB-3) for 8 min (3 mA), so that a thin layer of gold was deposited on the surface of the sample. The morphology of mycelia and conidia in three kinds of growth states (treated, recovered, and untreated) were observed by SEM and compared by measuring 10 mycelial diameter, branch number of 100 mycelial tips and 10 conidial dimensions in random. The experiment was carried out a total of twice.

### Pathogenicity of the treated pathogens

A method was designed to detect the pathogenicity of the treated and recovered *M*. *fructicola* and *P*. *digitatum* compared to normally growing cultures. The peach and citrus fruits were surface sterilized and wounded as described above. Since the treated and recovered fungi produced almost no conidia, a 2 mm plug from the edge of the untreated, treated or recovered colony was inoculated on one location of the three wounds on each fruit; the other two wounds were inoculated with 10 μL sterile water and non-inoculated controls. The infection number and lesion size of the two post-harvest diseases was measured 4 DPI. The pathogenicity test was conducted in a completely randomized design with three replicate boxes for each kind of inoculum with 10 fruits. This work was repeated.

### Statistical analysis

Data analysis was performed using Excel 2010 (Microsoft) and SPSS statistical program (Version 17.0, International Business Machines Corp., Armonk, New York). Mean values were compared using Student’s *t* test, at significance level α = 0.05.

## Results

### Effect of *C*. *fimbriata* volatiles on various fungi, oomycetes and bacteria

All test fungi, oomycetes and bacteria were greatly inhibited but not killed after exposure to volatiles from *C*. *fimbriata* in vitro without direct contact or diffusion through the culture media (Fig 1A). After 5 to 7 days treatment, the test fungi and oomycetes grew much more slowly than did untreated cultures. Inhibition of mycelial growth from 50% to 85% was observed (Table 2), which indicates that the sensitivity of each target microorganism to these volatiles varies.

**Table 2:** Effect of exposure to *Ceratocystis fimbriata* volatiles on the viability, mycelial growth, conidial production and spore germination of various plant pathogens.

Species	Viability ^x^	Inhibition of mycelial growth (%) ^y^	Conidial production (conidia/mL)		Inhibited percent (%) ^z^
			CK	Treated	
*Botrytis cinerea*	+	73±2	4×10^8^	0	80±3
*Monilinia fructicola*	+	65±1	5×10^6^	0	73±5
*Fusarium verticillioides*	+	49±2	3×10^7^	0	77±4
*Fusarium oxysporum*	+	50±2	3×10^7^	0	72±4
*Valsa mali*	+	54±2	NA	NA	NA
*Curvularia* sp.	+	64±3	3×10^5^	0	66±6
*Fusarium* sp.	+	60±2	4×10^7^	0	76±4
*Penicillium italicum*	+	ND	TNTC	0	87±3
*Penicillium digitatum*	+	84±2	9×10^11^	5×10^4^ *	69±3
*Rhizoctonia solani* (from rice)	+	79±2	NA	NA	NA
*Rhizoctonia solani* (from bent grass)	+	77±1	NA	NA	NA
*Phytophthora sojae*	+	75±1	NA	NA	NA
*Phytophthora capsici*	+	74±1	NA	NA	NA

+: Viable. TNTC = too numerous to count. NA = not applicable. ND = not determinable.

x = Viability after 5 to 7 days exposure to *C*. *fimbriata* when the diameter of the controls (untreated) reached about 8 cm.

y and z = mean value ± standard deviation (SD).

* indicates a significant difference between the control and the treatment by *C*. *fimbriata* volatiles (*P* < 0.05).

The colony morphology of treated fungi and oomycetes appeared abnormal, with thinner hyphae and lighter pigmentation (Fig 1B, 1E and 1H). In addition, some fungi, such as *P*. *italicum*, lost some pigmentation after the exposure to *C*. *fimbriata* volatiles (Fig 1G). For most of the test fungi, no conidia were observed after 5-day treatment by *C*. *fimbriata* VOCs, except *P*. *digitatum*, which produced 5×10^4^ conidia/mL (Table 2). The inhibition of mycelial growth and conidial production for each colony of *P*. *italicum* could not be calculated, since more than one colony usually formed due to conidial release (Fig 1G). Spore germination of test fungi was also affected by treatment by *C*. *fimbriata* VOCs, with observed germination inhibition in the range of 66% to 87% (Table 2). *Rhizoctonia* sp. belongs to mycelia sterile and does not produce any conidia. *V*. *mali* hardly produces conidia under normal conditions and only zoospores are produced by *Phytophthora* sp., so no data was collected on the effect of the treatment on conidial production of these species. The inhibition of mycelial growth on test fungi and oomycetes, except *P*. *italicum*, showed a linear relationship (correlated coefficient (R^2^) >0.9) with increasing incubation time and thus longer exposure to VOCs (Fig 1D). After exposure to *C*. *fimbriata* VOCs, all of the test bacteria were still alive, but none of them was able to produce normal discrete and round colony morphology; instead the colonies were irregularly shaped, and smeared to a bigger patchy zone (Fig 1I).

After the treatment by *C*. *fimbriata* volatiles, all test pathogens, including fungi, oomycetes and bacteria, grew after being transferred to a fresh plate. However, their growth speed was slow and their colony morphology abnormal after the 10^th^ generation (Fig 1C and 1F). The colony diameter of recovered pathogens growing on fresh plates was only 1.0 to 2.5 cm, significantly smaller than that of normally growing cultures (7.5 to 8.9 cm). No conidia were observed in most recovered cultures except in *P*. *digitatum*, whose conidial production and spore germination (2 × 10^4^ conidia/mL and 12 ± 1%, respectively) were much lower than that of the control (Table 3). This suggests that the treated pathogens cannot recuperate on fresh media, even when free from the exposure to the VOCs from *C*. *fimbriata*.

**Table 3:** Mycelial growth, conidial production and spore germination of the recovered pathogens growing on a fresh plate transferred from treated cultures.

Species	Diameter of colony (cm)^x^		Conidial production (conidia/mL)		Spore germination (%)^y^	
	CK	R	CK	R	CK	R
*Botrytis cinerea*	8.0 ± 0.2*	1.7 ± 0.1	4 × 10^8^ *	0	90 ± 2	ND
*Monilinia fructicola*	8.0 ± 0.1*	1.5 ± 0.1	5 × 10^6^ *	0	63 ± 4	ND
*Fusarium verticillioides*	8.0 ± 0.1*	1.8 ± 0.1	3 × 10^7^ *	0	76 ± 4	ND
*Fusarium oxysporum*	8.3 ± 0.2*	2.2 ± 0.2	3 × 10^7^ *	0	77 ± 4	ND
*Valsa mali*	8.1 ± 0.1*	2.2 ± 0.2	NA	NA	NA	NA
*Curvularia* sp.	7.5 ± 0.1*	1.8 ± 0.2	3 × 10^5^ *	0	69 ± 5	ND
*Fusarium* sp.	8.3 ± 0.1*	1.9 ± 0.1	4 × 10^7^ *	0	75 ± 4	ND
*Penicillium italicum*	ND	ND	TNTC	NA	89 ± 3	NA
*Penicillium digitatum*	7.8 ± 0.2*	1.8 ± 0.1	9×10^11^ *	2 × 10^4^	69 ± 1*	12± 1
*Rhizoctonia solani* (from rice)	8.5 ± 0.1*	1.6 ± 0.1	NA	NA	NA	NA
*Rhizoctonia solani* (from bent grass)	8.9 ± 0.1*	1.3 ± 0.1	NA	NA	NA	NA
*Phytophthora sojae*	7.9 ± 0.3*	2.2 ± 0.1	NA	NA	NA	NA
*Phytophthora capsici*	7.9 ± 0.1*	2.0 ± 0.1	NA	NA	NA	NA

x and y: expressed as mean value ± SD.

+: Viable. TNTC = too numerous to count. NA = not applicable. ND = not determinable.

* indicates a significant difference between the control and recovered treatment (P < 0.05).

### Control of peach brown rot and citrus green mold

**Fig 3:**
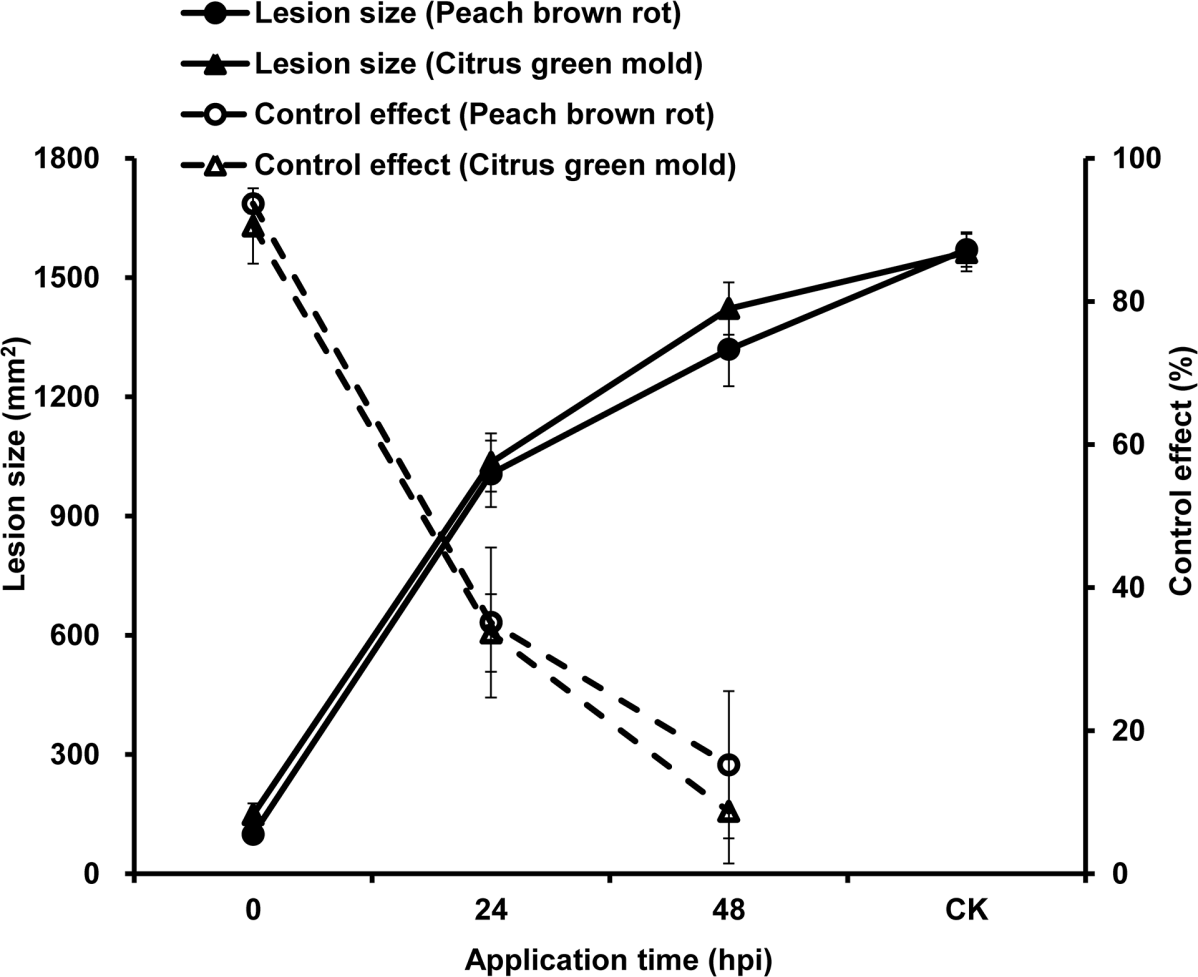
Effect of the treatment by the VOCs from *Ceratocystis fimbriata* applied immediately, 1 or 2 days after inoculation on the lesion size (mm^2^) and control effect (%) of peach brown rot and citrus green mold for four days. Error bars represent the standard deviation of ten replicates.

In the treatment without *C*. *fimbriata* volatiles (negative control), the fruits were 100% infected by *M*. *fructicola* or *P*. *digitatum* and visible rot or mold was observed on wound locations inoculated with conidial suspension (Fig 2C). In the treatments with *C*. *fimbriata* volatiles, no visible symptoms were observed on the fruits whether they were inoculated with conidial suspension or sterile water (Fig 2D and 2E). The disease control effect on peach brown rot and citrus green mold was 94% and 91% respectively when the VOCs treatment was performed immediately after inoculation with the conidia suspension, which was the optimum application time for the treatment; when treated 24 hours post inoculation (HPI) disease control sharply decreased to 35% and 34%, respectively. Disease control was only 15% and 9% respectively when treated 48 HPI for the two diseases (Fig 3).

**Fig 4:**
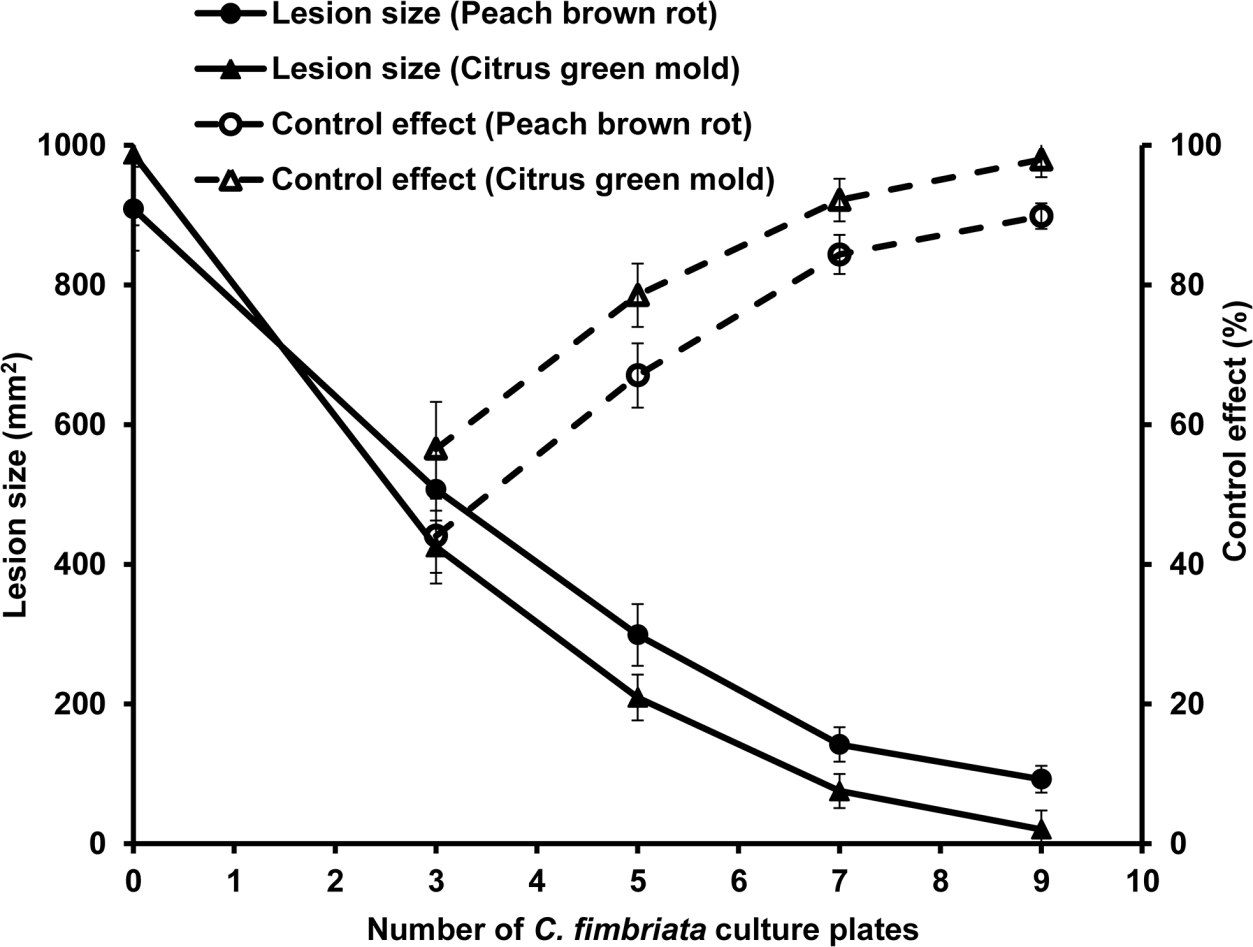
Lesion size (mm^2^) and control effect (%) of peach brown rot and citrus green mold treated by 0, 3, 5, 7, and 9 plates of *Ceratocystis fimbriata* culture 4 days post inoculation (DPI). Error bars represent the standard deviation of ten replicates.

The effect of VOCs dose on the two diseases was determined by fumigating the inoculated fruits with different amounts of *C*. *fimbriata* cultures in boxes for 4 days immediately after the inoculation. Untreated peaches and citrus were 100% infected, with lesion sizes of 909 mm^2^ and 987 mm^2^ respectively. Disease control for both of the plant diseases improved as the number of *C*. *fimbriata* cultures per box increased, with control as high as 90% and 98% with 9 plates and as low as 44% and 57% with three plates (Fig 4).

**Fig 5:**
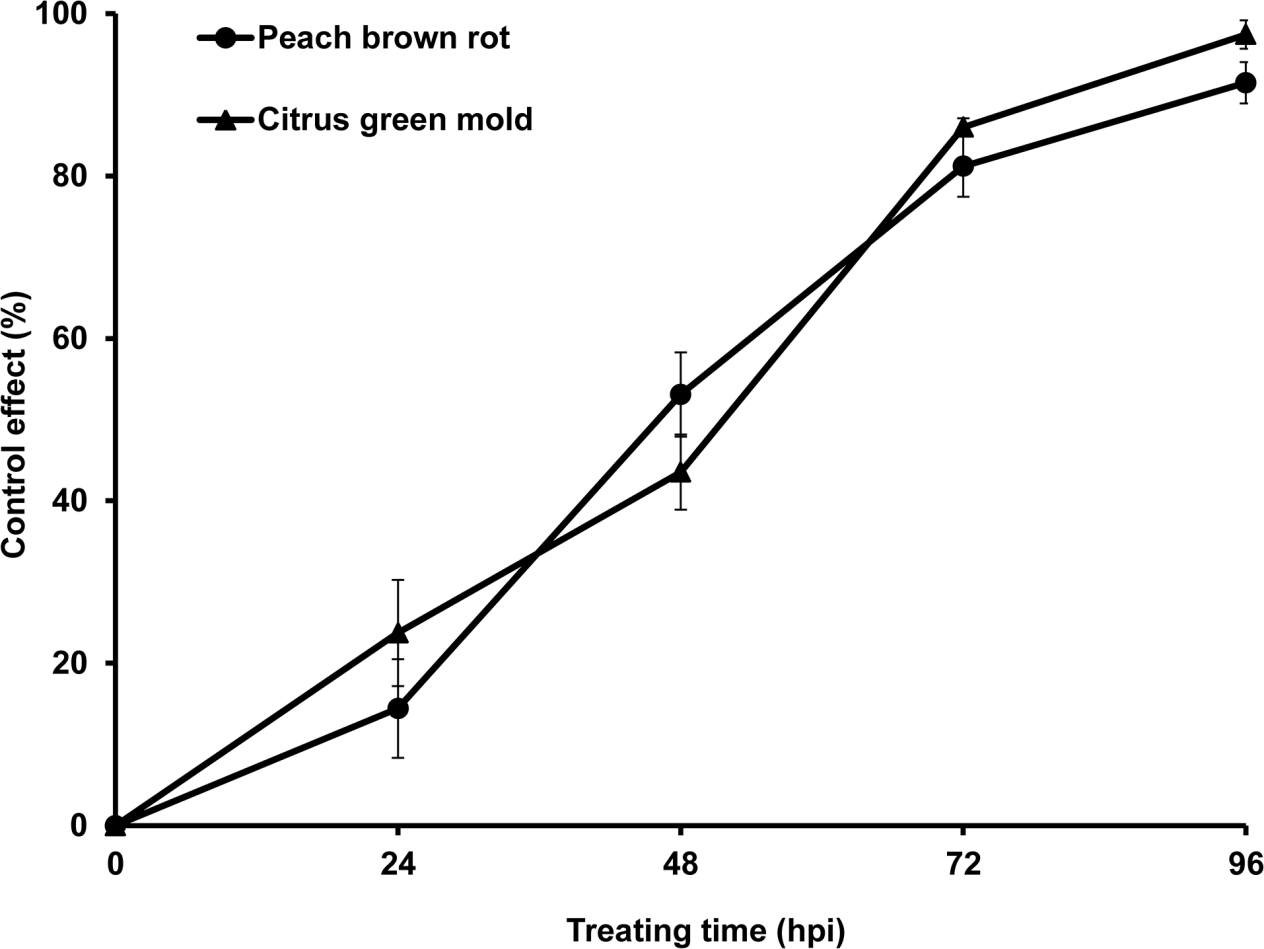
Control effect (%) on peach brown rot and citrus green mold 0, 24, 48, 72, and 96 hours post inoculation (HPI) treated by ten plates of *Ceratocystis fimbriata* cultures. Error bars represent the standard deviation of ten replicates.

Control effect treated by various treatment periods was tested with 10 *C*. *fimbriata* cultures in each box immediately after the inoculation. The control effect of peach brown rot and citrus green mold increased as the time of the treatment lasted longer, with control at 92% and 97% at 96 HPI (Fig 5), while the control fruits were completely infected.

### Morphological change of the treated pathogens

**Fig 6:**
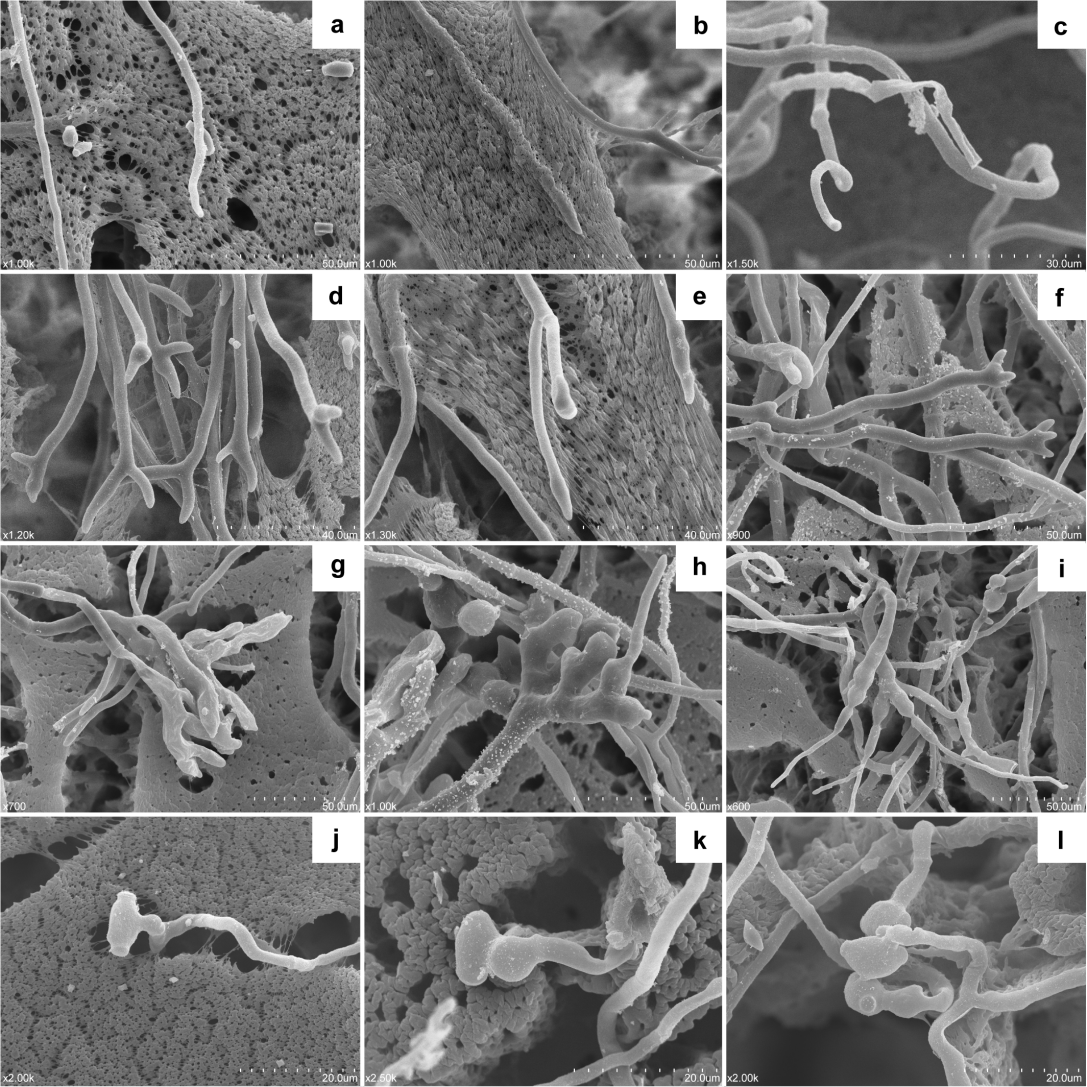
Hyphal and conidial morphology of *Monilinia fructicola* treated by the VOCs from *Ceratocystis fimbriata* observed by scanning electron microscope (SEM). (a) Mycelia of *M*. *fructicola* growing on PDA medium. (b-e) Misshapen mycelia of *M*. *fructicola* with 6-day exposure to the VOCs from *C*. *fimbriata*. (f-i) Recovered mycelia of *M*. *fructicola* was transferred to a fresh PDA plate after 6-day exposure to the VOCs from *C*. *fimbriata* and grown for another four days. (j) Conidia of *M*. *fructicola*. (k) Misshapen conidium of *M*. *fructicola* with 6-day exposure to the VOCs from *C*. *fimbriata*. (l) Recovered conidia of *M*. *fructicola* transferred to a fresh PDA plate after 6-day exposure to the VOCs from *C*. *fimbriata* and grown for another four days.

To evaluate the morphological change of the test pathogens treated by VOCs from *C*. *fimbriata*, the morphology of the treated mycelia and conidia was observed by SEM after 5 days treatment. In the control samples, *M*. *fructicola* grew healthily on PDA medium, presented regular and homogeneous mycelia with typical tapered apices (Fig 6A), and had normal barrel-shaped conidia (Fig 6J). Both hyphae and conidia had a smooth appearing cell wall surface. The treated samples exhibited abnormal growth with irregular distortions. The severely degenerating hyphae were collapsed, curling, distorted or twisted (Fig 6B and 6C), with swollen apexes with extensive branches (Fig 6D–6F), and the conidia were enlarged and distorted (Fig 6K). After recovering on a fresh PDA plate, *M*. *fructicola* still had a similarly misshapen morphology with that of the treated fungus; the hyphae and conidia were swollen, branched and twisted (Fig 6G–6I and 6L). Measurement results showed that the diameter of *M*. *fructicola* and *P*. *digitatum* control hyphae was significantly wider than that of treated and recovered treatment. The conidia size of *M*. *fructicola* in treated and recovered cultures was significantly larger than that in the control. However, there was no significant difference among the treated, recovered or normal *P*. *digitatum* cultures. One hundred mycelial tips were observed in each treatment for *M*. *fructicola* and *P*. *digitatum*, and almost all of the observed hyphae in the treated or recovered cultures were branched, compared to that with regular morphology in the control (Table 4).

**Table 4:** Effect of the exposure to *Ceratocystis fimbriata* on the morphology of *Monilinia fructicola* and *Penicillium digitatum* observed by SEM.

Test pathogens		CK ^x^	F (5 DPI) ^y^	R (5 DPI) ^z^
*M*. *fructicola*	Hyphal diameter (μm)	4.57 ± 0.19 a	3.43±0.20 b	3.46±0.20 b
	Branch number (/100 mycelia)	0 b	98 a	100 a
	Conidia size (μm)	(11.57±0.63) × (5.30±0.32) b	(15.85±0.31) × (8.49±0.25) a	(15.67±0.26) × (8.46±0.18) a
*P*. *digitatum*	Hyphal diameter (μm)	4.68±0.17 a	2.52±0.31 b	2.55±0.27 b
	Branch number (/100 mycelia)	0 b	97 a	99 a
	Conidia size (μm)	(4.54±0.29) × (3.29±0.18) a	(4.33±0.28) × (2.45±0.26) a	(4.35±0.17) × (2.49±0.25) a

Hyphal diameter (μm) and conidia size (μm) were expressed as mean value ± SD.

^x^ CK = Growing normally.

^y^ F = Treated, 5-day exposure to *C*. *fimbriata* volatile compounds.

^z^ R = Recovered, transferred to a fresh PDA plate after 5-day exposure to *C*. *fimbriata* volatiles.

a and b represent statistically significant differences between the control, treated and recovered treatment (p < 0.05).

### Pathogenicity of the treated pathogens

The loss of pathogenicity determinants was another possible reason for the bioactivity of *C*. *fimbriata* VOCs on the test pathogens. In the control, all of the fruits inoculated with normal *M*. *fructicol*a or *P*. *digitatum* were 100% infected with typical symptoms of peach brown rot and citrus green mold. However, no visible symptoms were observed on the fruits inoculated with treated or recovered pathogens (Fig 2F). A plug from the colony edge of *M*. *fructicola* treated with the *C*. *fimbriata* volatiles was transferred to the 10^th^ generation. The colony grew, but the growth rate was slow, and had the similar colony morphology compared to the first generation of treated culture. It is possible that the pathogenicity of *M*. *fructicola* and *P*. *digitatum* was lost or weakened after being treated by *C*. *fimbriata* VOCs due to damage to cell wall or organelles, which cannot recuperate when the treated pathogens were transferred to fresh culture media.

## Discussion

VOCs from *C*. *fimbriata* have significant inhibition on the test fungi, oomycetes and bacteria. Misshapen mycelia and conidia were observed by SEM after treated with VOCs. These prominent morphological changes in treated samples were possibly due to the damage of the cell wall or the lack of cytoplasm [28, 29]. And it also might be from the destruction of organelles in the endomembrane system [30]. Morphological alterations and damage on the hyphae or conidia were the possible reasons for the slow mycelial growth speed and low conidial germination. VOCs also inhibited pigment production, such as in *Fusarium* sp., which was also observed in other treated fungi by VOCs from bacteria [31]. Interestingly, the changed characteristics affected by VOCs remained even after 10 generations of transfer. If the morphological changes are carried over the generations, it is possible that genetic mutations happened. However, as we did not conduct further studies, it is too early to make such a conclusive statement, but is definitely worth to examine in future work.

Exposure to VOCs from *C*. *fimbriata* showed significant inhibition of test fungi, oomycetes and bacteria *in vitro* and excellent control of peach brown rot and citrus green mold. Unlike some traditional biological control agents, which must colonize wounds or some other susceptible sites to be effective [32], the VOCs from *C*. *fimbriata*, acting as a biofumigant, does not require contact. Such treatment may be more compatible with integrated disease or pest management systems than current biological or chemical fungicides that require spraying or drenching applications, causing problems such as fungicide residue, air pollution, and water purification [21]. The VOCs from wild-type strain *F*. *oxysporum* MSA 35 repressed gene expression of two virulence genes in *F*. *oxysporum* f. sp. *lactucae* [33]. It can be deduced that there also might be some genes associated with pathogenicity affected by VOCs from *C*. *fimbriata*.

We have qualitatively and quantitatively determined the VOCs from *C*. *fimbriata* by headspace GC-MS (unpublished data). Seven compounds were identified, and the three most abundant were butyl acetate, ethyl acetate and ethanol, which accounted for 97% of the total VOCs yield. Mixtures of one or more of these pure chemicals were used to simulate the treatment on the test pathogens at the approximate concentration of VOCs naturally produced by *C*. *fimbriata*, but there was no obvious inhibition effect observed. Inhibition may be due to the synergistic effect of all VOCs from *C*. *fimbriata*, including those that cannot be detected by current identification methods. This is similar to results from other VOCs producing fungi, such as *Muscodor* sp. [21, 34]. Since *C*. *fimbriata* is also a soil-borne pathogen, the authors proposed that the practical application could be to screen a mutant without pathogenicity or to incubate this fungus in a container and transfer the VOCs produced into a separate airtight container by pump, which should avoid the risk of possible infection by *C*. *fimbriata* by direct contact with agricultural products or through the soil.

There are few studies on antimicrobial volatiles from *C*. *fimbriata*, and to the best of our knowledge, this is the first report on the potential of *C*. *fimbriata* against other plant pathogens and plant diseases control. The bioactivity of VOCs from *C*. *fimbriata* suggested that this biofumigation could be applied for diseases control under airtight conditions, not only on fresh fruits but also in cereals, vegetables, seeds, and seedlings.

## Supporting Information

S1 Fig Morphology of *Ceratocystis fimbriata*. **(**a) colony morphology. (b) perithecia and ascospore masses. (c) perithecia with an ascospore mass. (d) perithecia with a globose base. (e) divergent ostiolar hyphae with ascospores emerging through the mouth of the neck. (f) hat-shaped ascospores. (g) barrel-shaped conidia. (h) conidiophore with cylindrical conidia released from the phialide. (i) cylindricalconidia. (j) aleuroconidia. Scale bar for b = 500 μm, scale bar for d = 100 μm, scale bars for e-j = 10 μm.
(TIF)
