# Supplementary figures and images for: Biofumigation on Post-Harvest Diseases of Fruits Using a New Volatile-Producing Fungus of Ceratocystis fimbriata
Source: PLoS One. 2015 Jul 6;10(7):e0132009. doi: 10.1371/journal.pone.0132009 (PMC4492557; doi:10.1371/journal.pone.0132009)

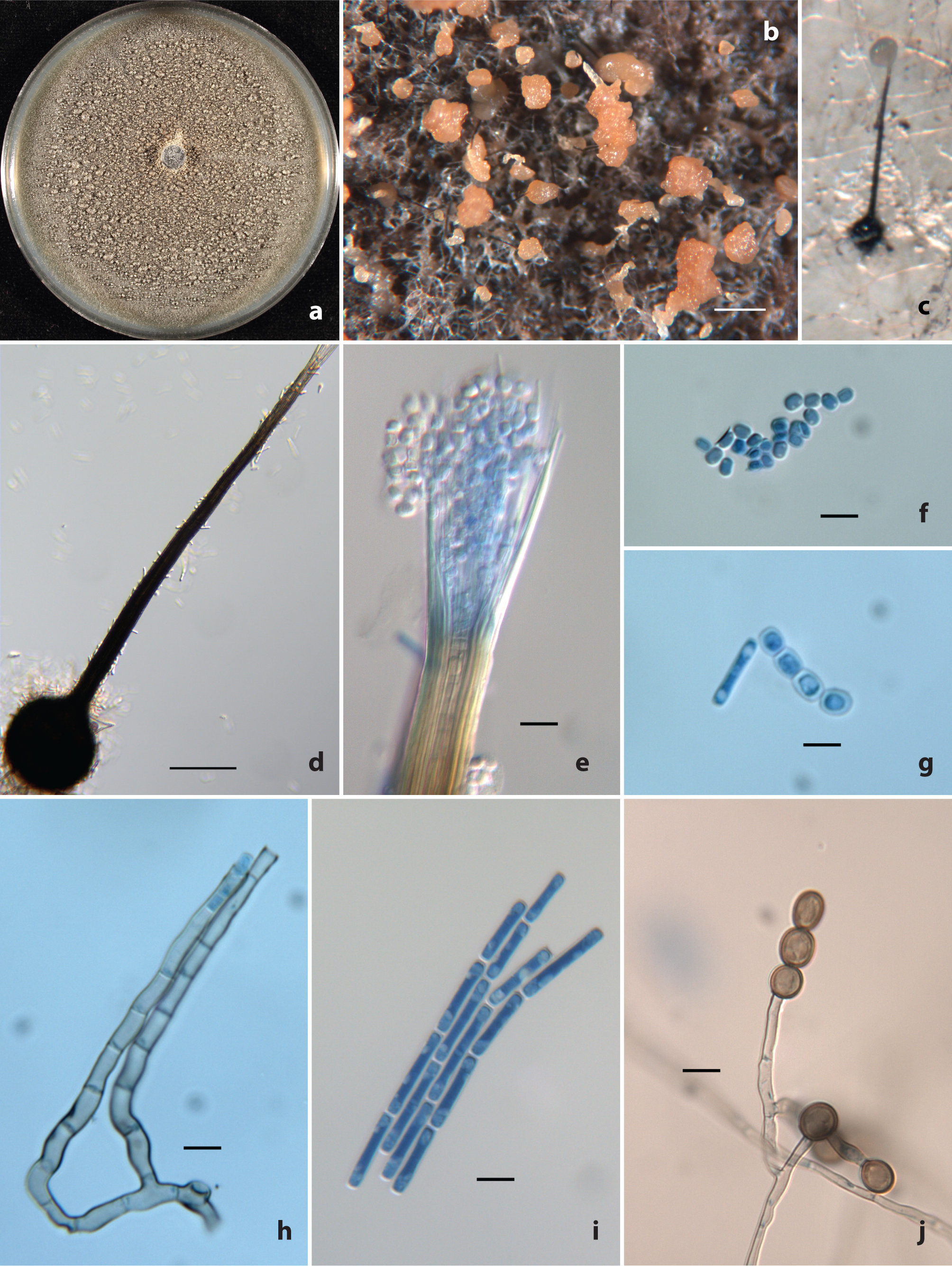

Supplement: S1 Fig — (a) colony morphology. (b) perithecia and ascospore masses. (c) perithecia with an ascospore mass. (d) perithecia with a globose base. (e) divergent ostiolar hyphae with ascospores emerging through the mouth of the neck. (f) hat-shaped ascospores. (g) barrel-shaped conidia. (h) conidiophore with cylindrical conidia released from the phialide. (i) cylindricalconidia. (j) aleuroconidia. Scale bar for b = 500 μm, scale bar for d = 100 μm, scale bars for e-j = 10 μm. (TIF) [file pone.0132009.s001.tif]
